# Modelling the historical distribution of schistosomiasis-transmitting snails in South Africa using ecological niche models

**DOI:** 10.1371/journal.pone.0295149

**Published:** 2023-11-30

**Authors:** Nisa Ayob, Roelof P. Burger, Monray D. Belelie, Ncobile C. Nkosi, Henno Havenga, Lizaan de Necker, Dirk P. Cilliers

**Affiliations:** 1 Unit for Environmental Sciences and Management, North-West University, Mafikeng Campus, Mafikeng, South Africa; 2 Unit for Environmental Sciences and Management, North-West University, Potchefstroom Campus, Potchefstroom, South Africa; 3 South African Institute for Aquatic Biodiversity (NRF-SAIAB), Makhanda, South Africa; 4 Water Research Group, Unit for Environmental Sciences and Management, North-West University, Potchefstroom, South Africa; Penn State University, UNITED STATES

## Abstract

Schistosomiasis is a vector-borne disease transmitted by freshwater snails and is prevalent in rural areas with poor sanitation and no access to tap water. Three snail species are known to transmit schistosomiasis in South Africa (SA), namely *Biomphalaria pfeifferi*, *Bulinus globosus* and *Bulinus africanus*. In 2003, a predicted prevalence of 70% was reported in tropical climates in SA. Temperature and rainfall variability can alter schistosomiasis-transmitting snails’ development by increasing or decreasing their abundance and geographical distribution. This study aimed to map the historical distribution of schistosomiasis from 1950 to 2006 in SA. The snail sampling data were obtained from the historical National Snail Freshwater Collection (NFSC). Bioclimatic variables were extracted using ERA 5 reanalysis data provided by the Copernicus Climate Change Service. In this study, we used 19 bioclimatic and four soil variables. The temporal aggregation was the mean climatological period pre-calculated over the 40-year reference period with a spatial resolution of 0.5° x 0.5°. Multicollinearity was reduced by calculating the Variance Inflation Factor Core (VIF), and highly correlated variables (> 0.85) were excluded. To obtain an "ensemble" and avoid the integration of weak models, we averaged predictions using the True Skill Statistical (TSS) method. Results showed that the ensemble model achieved the highest Area Under the Curve (AUC) scores (0.99). For *B*. *africanus*, precipitation-related variables contributed to determining the suitability for schistosomiasis. Temperature and precipitation-related variables influenced the distribution of *B*. *globosus* in all three models. *Biomphalaria pfeifferi* showed that Temperature Seasonality (bio4) contributed the most (47%) in all three models. According to the models, suitable areas for transmitting schistosomiasis were in the eastern regions of South Africa. Temperature and rainfall can impact the transmission and distribution of schistosomiasis in SA. The results will enable us to develop future projections for *Schistosoma* in SA based on climate scenarios.

## Introduction

Schistosomiasis is a parasitic infection from infestation by trematode worms belonging to the genus *Schistosoma* [1]. This disease is spread through vectors and is most common in 78 countries, mainly tropical and subtropical regions. After malaria, schistosomiasis is the second most impactful disease regarding socioeconomic impact on affected individuals [1–3]. Sub-Saharan Africa is one of the most endemic areas for this disease, with 800 million people infected and 200 to 535 thousand deaths annually [3]. Schistosomiasis is widespread due to unsanitary conditions, inadequate sanitation, and a lack of clean water [4]. Among poor rural populations, most cases are linked to agriculture and fishing [5]. During their daily activities, fishermen and farmers are in contact with snail-infested water, making them susceptible to schistosomiasis [6]. Communities that do not have access to tap water are more likely to contract the disease since they travel to snail-infested water sources for domestic activities such as laundry, bathing, and cooking [5]. Freshwater snails are the intermediate hosts that transmit schistosomiasis to people and livestock. The main parasites known to infect humans are *Schistosoma haematobium* and *Schistosoma mansoni* [6]. *Schistosoma*. *haematobium*, causing urogenital schistosomiasis, and is transmitted by *B*. *globosus* and *B*. *africanus*. *Schistosoma mansoni* causes rectal and intestinal schistosomiasis, affecting the intestines and transmitted by *B*. *pfeifferi*. The parasites commonly inhabit areas where domestic and agricultural activities occur.

Approximately 5 million South Africans living in poor rural areas lack access to clean water [7]. In rural areas without running water, communities near water bodies (rivers, ponds, lakes, and dams) often become infected with schistosomiasis [8]. The prevalence of schistosomiasis ranges between 7.7% and 70% among school-going children, who appear to have the highest prevalence of this infection [8, 9]. Schistosomiasis occurs in areas where snail vectors have suitable habitats. The suitable habitats where the snail vectors thrive are tropical, humid, and aquatic environments such as dams and rivers [10]. These intermediate host snails thrive in regions with mild to warm temperatures (15–25°C) and moderate to heavy rainfall events (300-900mm/a) [10, 11]. The most prevalent geographical areas in SA include the majority of Mpumalanga, Limpopo and the coast of KwaZulu-Natal and the eastern regions of Gauteng and North West provinces [11–14]. Although intermediate host snails reproduce through aestivating during dry seasons, snails are limited to areas with enough humidity for survival [14].

During the past decade, climate variability has led to increasing schistosomiasis cases in Africa, with an estimated 90% of infections [15]. In addition, studies suggest that temperature and precipitation have contributed to changes in water availability [16, 17]. As temperatures increase, the water in waterbodies can become stagnant and provide a breeding ground for the parasites that cause the disease [16–18]. Shifts in precipitation patterns, rising temperatures, frequent droughts and floods may impact the intermediate host snails [17–19]. As environmental conditions become favourable, parasites and snails can spread into areas that were not previously endemic [20]. Climate variability can affect water flow, temperature, and precipitation, which affect vectors’ behavioural and geographical patterns [16]. *Bulinus africanus* is primarily distributed in the eastern regions of SA, while *B*. *globosus* is prevalent in the northeastern areas of Limpopo and Mpumalanga. Due to its sensitivity, *B*. *pfeifferi* is unlikely to be present in temporary rainy habitats [21]. These species typically live in still or slow-moving permanent waterbodies. Similar to *B*. *africanus*, *B*. *pfeifferi* distribution covers most of SA and is influenced by the water quality of water bodies and temperature [22]. De Kock et al. [23] studied *Bulinus* species distribution and habitats in SA using decision trees and found that temperature is the most crucial factor determining the geographic range for *B*. *africanus*. It was supported by Brown’s findings [24], showing that *B*. *africanus* is associated with cooler weather than *B*. *globosus*.

In recent years, ecological niche modelling (ENM) has become increasingly popular [25–27]. In addition to predicting distributional ranges, the model can identify which climatic and environmental variables shape the distribution of species [26]. Ecological niche modelling has many approaches, each producing a unique prediction and map using bioclimatic data [26, 28–30]. Studies in SA have previously applied the ENMs [31] and China [32] to estimate snail habitats as intermediate hosts for schistosomiasis transmission. However, no single approach is most effective for modelling the distribution of schistosomiasis, as discussed by [33, 34].

In SA and Ethiopia, urogenital schistosomiasis is more common due to cooler climates than in other African countries [4]. South Africa is among the most vulnerable countries in the world owing to these climatic changes, especially given the high incidence of several life-threatening diseases, poverty, and unequal access to health care. Schistosomiasis may spread to previously unaffected areas with the spread of *Schistosoma* parasites in warmer waters [5, 9, 35]. Climate change alters distribution patterns and the geographical distribution of schistosomiasis [20]. There have been few attempts to model the historical distributions of *Schistosoma* transmitting snails in SA using environmental variables. Therefore, this study aimed to use ecological niche models (ENMs) to simulate historical distributions of infected snails in SA to fill this knowledge gap. In this paper, we modelled all three vectors of schistosomiasis: *B*. *africanus*, *B*. *globosus* and *B*. *pfeifferi*. These maps will enable us to accurately forecast the spread of schistosomiasis in South African endemic areas and aid in strategically planning preventive measures.

## Materials and methods

### Study area

**Fig 1:**
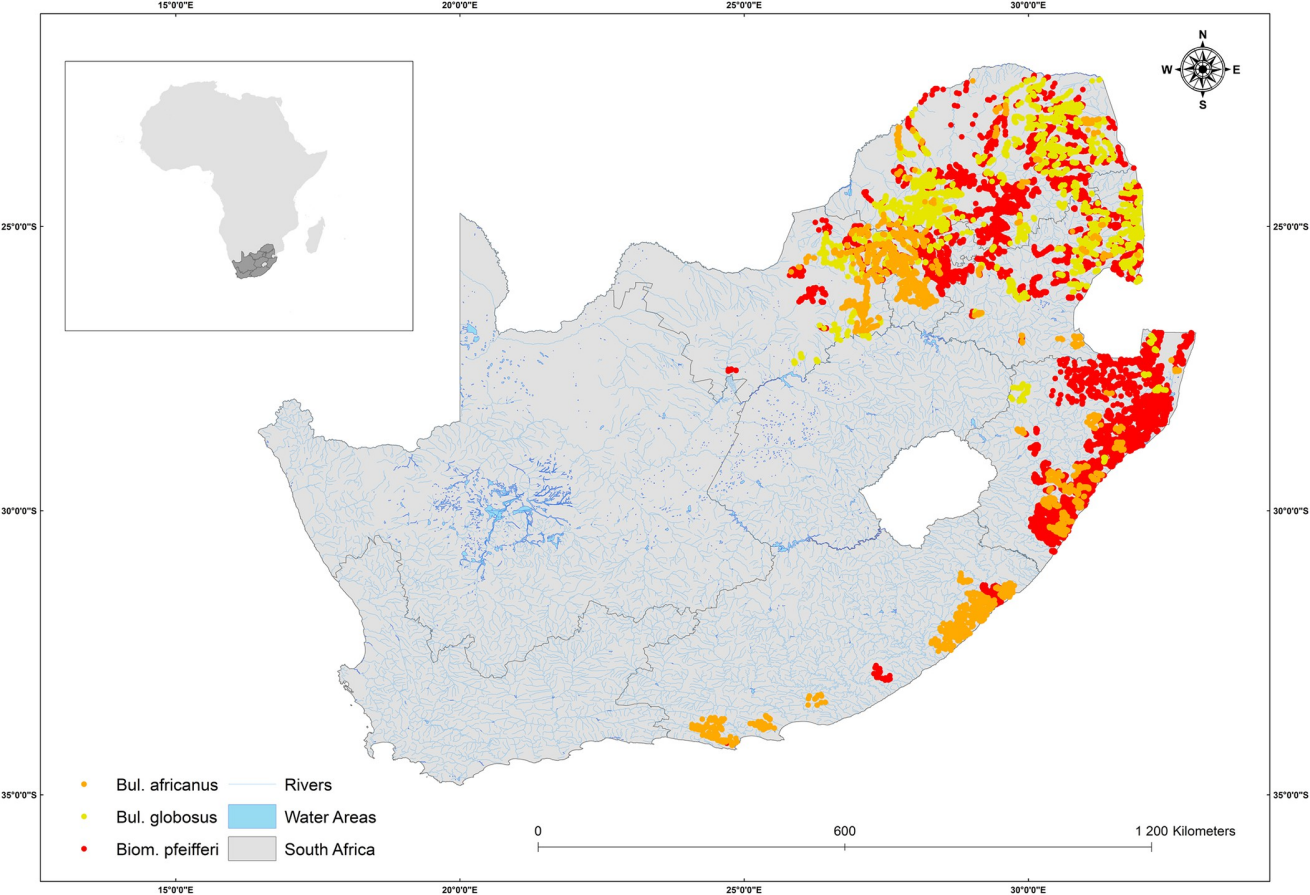
Map of South Africa illustrating snail vectors of schistosomiasis species. The orange points show *B*. *africanus*, the yellow depicts *B*. *globosus*, and the red represents *B*. *pfeifferi*.

South Africa’s surface area covers 1 219 602 km^2^. It extends along its latitudes from 22°S to 35°S as well as along its longitudinal axis from 17°E to 33°E [36, 37] (Fig 1). From the 1950s to 2006, snails infected with parasites were historically sampled from rivers and dams in SA. The country receives about 464 mm of rainfall annually [36]. Rainfall patterns are changing, resulting in severe storms, landslides, and flash flooding in the eastern regions of SA. Studies have shown that floods can lead to schistosomiasis outbreaks [38, 39]. During floods, people are exposed to contaminated water, increasing their risk of schistosome infection. Temperatures in SA have increased steadily over the last 60 years, impacting the survival and distribution of intermediate host snails [40–42]. A study conducted in Limpopo Province found that winters are becoming wetter and warmer, and summers are getting drier and warmer [43]. A typical summer temperature in SA ranges from 15°C to 36°C and a winter temperature of -2°C to 26°C [36, 40]. As temperatures increase, people with inadequate housing and access to clean water will likely become more vulnerable to schistosomiasis.

### Occurrence data

The occurrence data were obtained from the historical National Snail Freshwater Collection (NFSC) from 1950 to 2006, and 15303 records were recorded. The occurrence dataset for *B*. *africanus*, *B*. *globosus* and *B*. *pfeifferi* is illustrated in Table 1. The original sampling points were stored in quarter-degree grids. The snail sampling points were refined by digitising based on the descriptions of the sampling points on a 1:50000 topographical scale using ArcGis 10.8.2. The locations were digitised close to waterbodies (dams, rivers, reservoirs, lakes and swamps). Latitude and longitude coordinates were stored in decimal degrees using a WGS84 datum. South African administrative boundaries and hydrological data were downloaded from DIVA-GIS (https://www.diva-gis.org/gdata). The African shapefile was downloaded from Natural Earth (https://wwwnaturalearthdata.com/).

**Table 1:** The number of historical data points for snail vectors of schistosomiasis species found in South Africa from 1950–2006.

Species	Occurrence Points
*Bulinus africanus*	3051
*Bulinus globosus*	3153
*Biomphalaria pfeiferri*	9166

### Environmental variables

From 1970 to 2018, bioclimatic variables were extracted using ERA 5 reanalysis data provided by the Copernicus Climate Change Service. This study used 19 bioclimatic and four soil variables from 1970 to 2006. These indicators describe how climate affects species’ habitats and can be used for biodiversity applications. The temporal aggregation was the mean climatological period pre-calculated over the 40-year reference period 1979–2018 with a spatial resolution of 0.5° x 0.5°. The data was downloaded as NetCDF-4 files and was converted to a raster using multidimensional tools in ArcGIS 10.8.2. The data were prepared in ArcGIS and RStudio 4.2.1.

### Selection of environmental variables

A group of variables was chosen based on bioclimatic and soil factors to minimise collinearity in the dataset shown in Table 2. The *USDM* R package calculated the Variance Inflation Factor Core (VIF). This analysis measures the strength of the relationship between each predictor and the rest. In VIFs, the multiple correlation coefficients (R2) are obtained by performing a regression analysis of each predictor variable against the other variables in the model. As a rule of thumb, a VIF >10 indicates a collinearity problem [44]. All the variables >10 were removed from the dataset using a threshold (th) of 0.85. The VIF is calculated by excluding highly correlated variables through a stepwise procedure Table 2. During the VIF calculation process, the algorithm looks for variables with a linear correlation exceeding the predefined th value. It was done for the averages over the period 1970–2006. The process was repeated until none of the variables had a high correlation coefficient (r). To determine VIF, a linear regression model was employed, where the numerical variable of interest was utilised as the response variable Eq (1). The final set of bioclimatic predictors is illustrated in Table 2. It must be noted that, due to the nature and distribution of the species, each species has a unique set of environmental variables.


$$VIF=\frac{1}{1-R_{i}^{2}}$$ (1)

Where R2, represents the linear model’s regression coefficient.

**Table 2:** The selection of bioclimatic variables for the ecological models based on their VIF values.

Species	Variable	Description	VIF
*B*. *africanus*	Bio 3 Bio 5 Bio 8 Bio 13 Bio 15 Bio 16 Soil water volume for the wettest quarter Soil water volume for the coldest quarter Soil water for the warmest quarter	Isothermality Max temperature of the warmest month Precipitation of the wettest month Precipitation seasonality Precipitation of the wettest quarter	9.1 4.0 4.1 5.5 6.6 7.4 9.0 6.3 5.5
*B*. *globosus*	Bio 4 Bio 7 Bio 8 Bio 15 Bio 16 Bio 17 Soil water for the warmest quarter	Temperature seasonality Temperature Annual Range The mean temperature of the wettest quarter Precipitation seasonality Precipitation of the wettest quarter Precipitation of the driest quarter	3.9 3.1 3.8 3.2 5.8 5.7 1.7
*B*. *pfeifferi*	Bio 4 Bio 8 Bio 12 Bio 16 Bio 18	Temperature seasonality The mean temperature of the wettest quarter Annual precipitation Precipitation of the wettest quarter Precipitation of the warmest quarter	6.9 3.2 7.3 9.2 3.1

### Modelling procedures and data analysis

Three ENMs were applied in this study to overcome their limitations: Generalized Linear Model (GLM), Maximum Entropy (MaxEnt) and Random Forest (RF), as illustrated in Table 3. This study chose logistic regression since it is the most widely used GLM form for environmental modelling. MaxEnt estimates the distribution within the investigation area based on the current locations’ environmental conditions. Using the distribution area, it selects an area with the maximum entropy [45]. RF consists of each tree constructed using a random subset of predictor variables. As a result, decor-related trees are created and reduce the model variance [46–48].

**Table 3:** GLM, RF, and MaxEnt characteristics used in the SDM.

ENM	Method	Format	Reference
GLM	Regression analysis	Presence/Pseudo-absence	[47]
RF	Regression analysis	Presence/Pseudo-absence	[48]
MaxEnT	Maximum Entropy	Presence/ Pseudo-absence	[25]

The present study is based on a multi-model SDM approach [49] implemented in the SDM package, and *dismo* was used to examine and model species distribution [49]. This R package unifies different implementations of SDM into one object-oriented framework that is reproducible and extensible. Based on the methodology of [50], the occurrence data were divided into two groups. Models were evaluated using 70% training data and 30% test data. The analysis was conducted with 5000 random pseudo-absence points. While presence-only models are commonly used, ENM model evaluations show that presence-background techniques are more efficacious [51]. To evaluate the stability and accuracy of the models, the number of maximum iterations was increased to 5,000 iterations using subsets to reduce underestimations and overestimations. By doing so, the model will have adequate time to converge. The regularisation remained at 1 to reduce model overfitting [51, 52].

Higher values in the area under the curve (AUC), Relative Operating Characteristics (ROC) and True Skill Statistics (TSS) indicated a better performance [53] in evaluating the model’s predictions. Known as the Hanssen Kuipers Discriminator, TSS compares the number of actual positive forecasts to the number of correct hypothetical projections. It is pertinent to note that TSS includes omissions and commissions. A value of +1 indicates accurate classification. In contrast, a value of -1 indicates no better performance than random. AUC can be used to calculate ROC values. Random predictions are represented by 0.5, and predictions > 0.5 are better than the random model [54, 55]. The AUC values analyse the presence and absence of data across a range of thresholds. There are four levels of AUC: 0 (unsuitable), 0.7–0.8 (suitable), 0.8–0.9 (highly suited) and >0.9 (extremely suited) [53].

Based on the results of three models, an ensemble model was developed to model the distribution of schistosomiasis in SA. Ensemble models combine all algorithms, which improves performance [53, 56]. All the models generated TSS, as shown in Table 4. Based on this, to create an ensemble, all three models were combined using "Weighted averaging” by using the TSS that was > 0.75 [56]. The weighted averaging gives more weight to the model with higher accuracy than other techniques.

**Table 4:** AUC and TSS for *B*. *africanus*, *B*. *globosus* and *B*. *pfeifferi* for the different models used in this study.

Species	Model	AUC	TSS
*Bulinus africanus*	GLM	0.87	0.7
	MaxEnt	0.95	0.77
	RF	0.95	0.93
	Ensemble	0.99	0.95
*Bulinus globosus*	GLM	0.94	0.78
	MaxEnt	0.95	0.79
	RF	0.98	0.89
	Ensemble	0.99	0.94
*Biomphalaria pfeifferi*	GLM	0.92	0.73
	MaxEnt	0.93	0.75
	RF	0.97	0.83
	Ensemble	0.99	0.94

## Results and discussion

### Performance of the models

Table 4 illustrates the AUC and TSS values for the different models and species. All three models had significant AUC and TSS values, demonstrating excellent performance. The RF outperformed the other models in terms of model performance across different species, followed by MaxEnt and GLM. The results of Wouyou et al. [55] were similar, with RF outperforming four ecological models.

Results showed that the ensemble models achieved the highest AUC scores (0.99) among the three ENM models. Evaluation of the model showed that it was based on competence rather than chance in modelling the historical distribution of schistosomiasis. It must be noted that the high AUC value provides confidence that the ensemble model can be used to examine schistosomiasis-prone areas under current and future climates.

**Fig 2:**
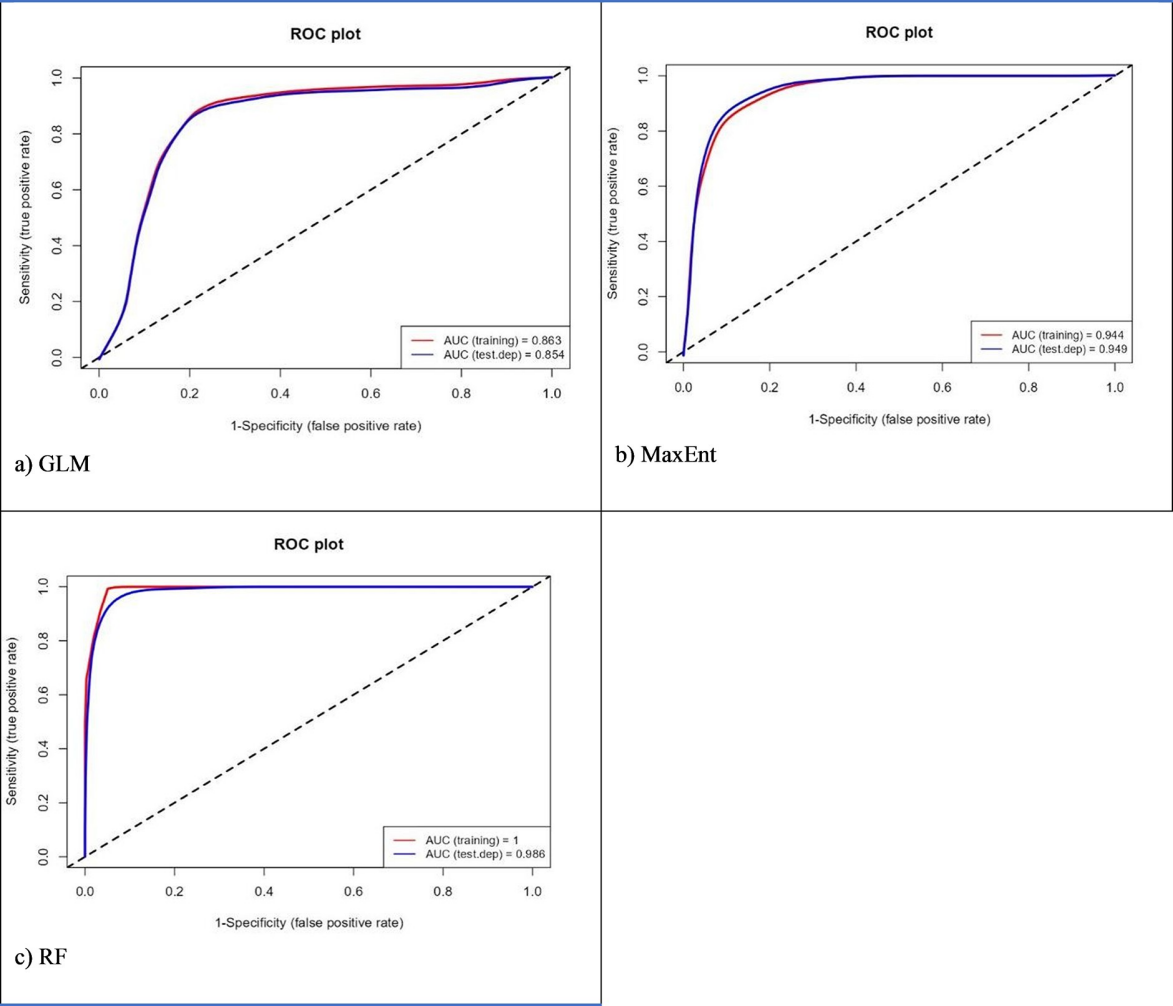
Area under the curve and Relative Operating Characteristics for *B*. *africanus* using three different models, namely (A) GLM, (B) MaxEnt, and (C) RF.

**Fig 3:**
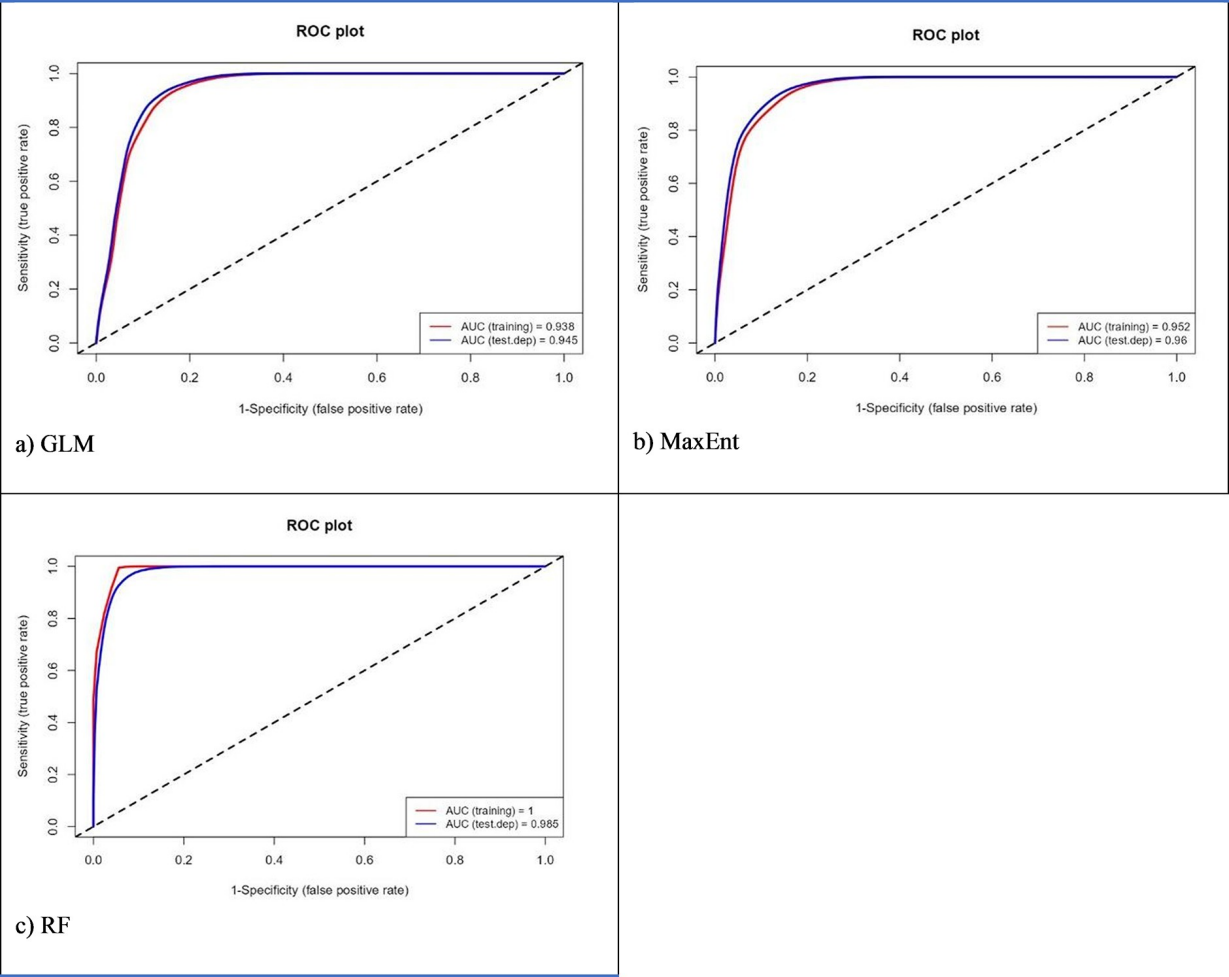
Area under the curve and Relative Operating Characteristics for *B*. *globosus* using three different models, namely (A) GLM, (B) MaxEnt, and (C) RF.

**Fig 4:**
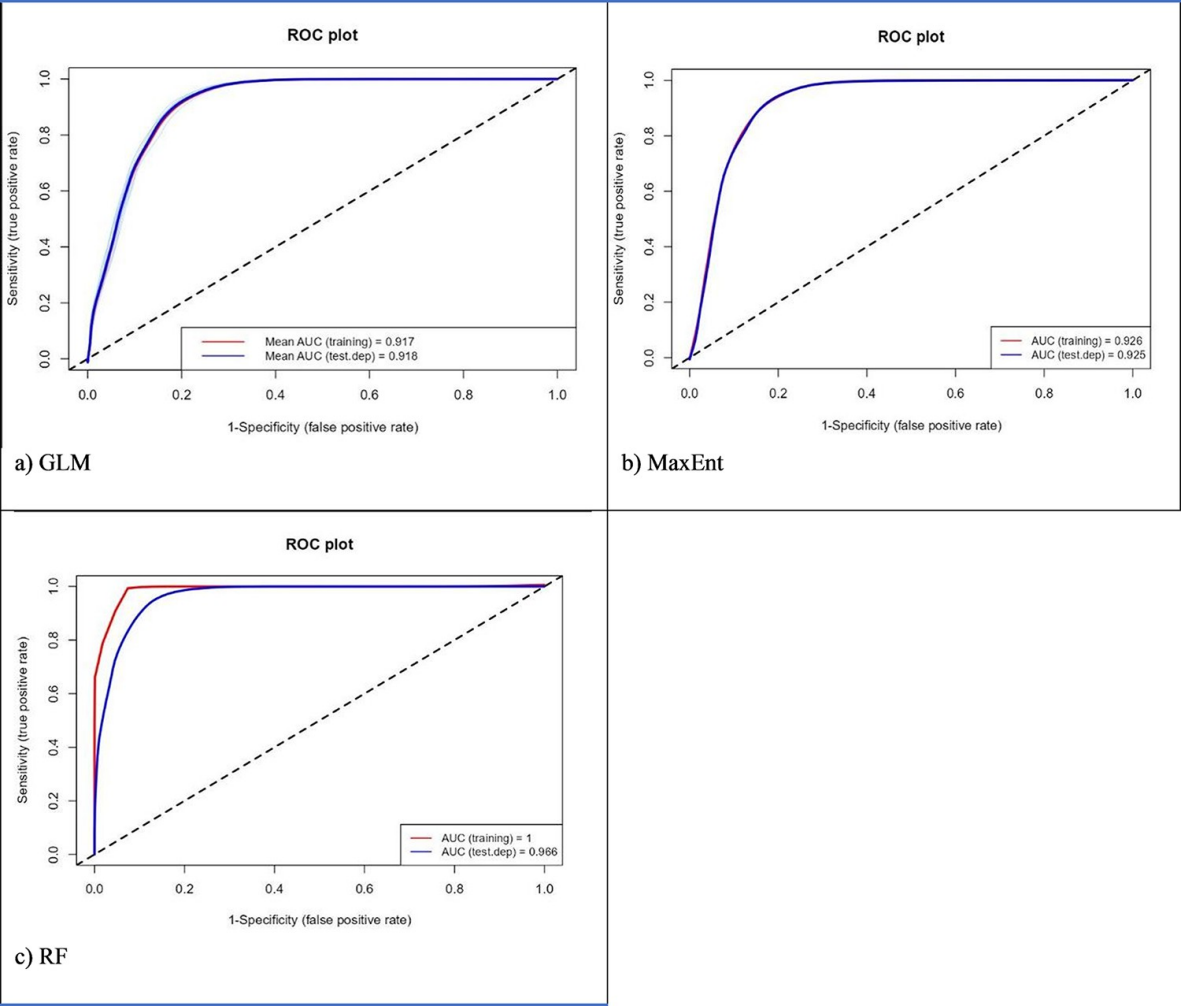
Area under the curve and Relative Operating Characteristics for *B*. *pfeifferi* uses three different models, namely (A) GLM, (B) MaxEnt, and (C) RF.

Figs 2–4 illustrate ROC plot graphs for *B*. *africanus*, *B*. *globosus* and *B*. *pfeifferi*. These figs show a range of threshold probability values for the True Positive Rate (sensitivity) and False Positive Rate (1-specificity) on the Y-axis. The closer ROC follows sensitivity, the more AUC will have a larger area. A ROC curve that follows the Y-axis closely, the AUC has a larger size. Thus, the model will be more accurate. A random model or guess will result in a point along the grey-dotted diagonal line. The results showed that the average ROC scores for GLM, MaxEnt and RF performed well, with all the models averaging an ROC score ≥ 0.89. For all three models, RF performed the most accurately (1), followed by MaxEnt (0.93) and GLM (0.9). This indicates that RF is the most accurate model for predicting species occurrence. Furthermore, the high AUC scores suggested that the models can be used to predict species occurrence with a high degree of confidence.

All three models performed well in the historical distribution areas of *B*. *africanus*, *B*. *globosus* and *B*. *pfeifferi*. Based on its accuracy in determining species distribution limits, the RF model performed better than MaxEnt and GLM. The RF model produced the most accurate results than the ensemble model. The results from the present study were similar to those [57]. They found RF, followed by MaxEnt, the best model for modelling Egypt’s medicinal species. The results from the current study were like those obtained by [58] when they compared ENMs Genetic Algorithm for Rule Set Production (GARP), MaxEnt, and GLM. MaxEnt was not considered the most effective model based on performance in this study. Nevertheless, it is widely acknowledged to have a robust predictive capability in conservation studies [57, 59–61].

### Variable contribution to the different models

**Fig 5:**
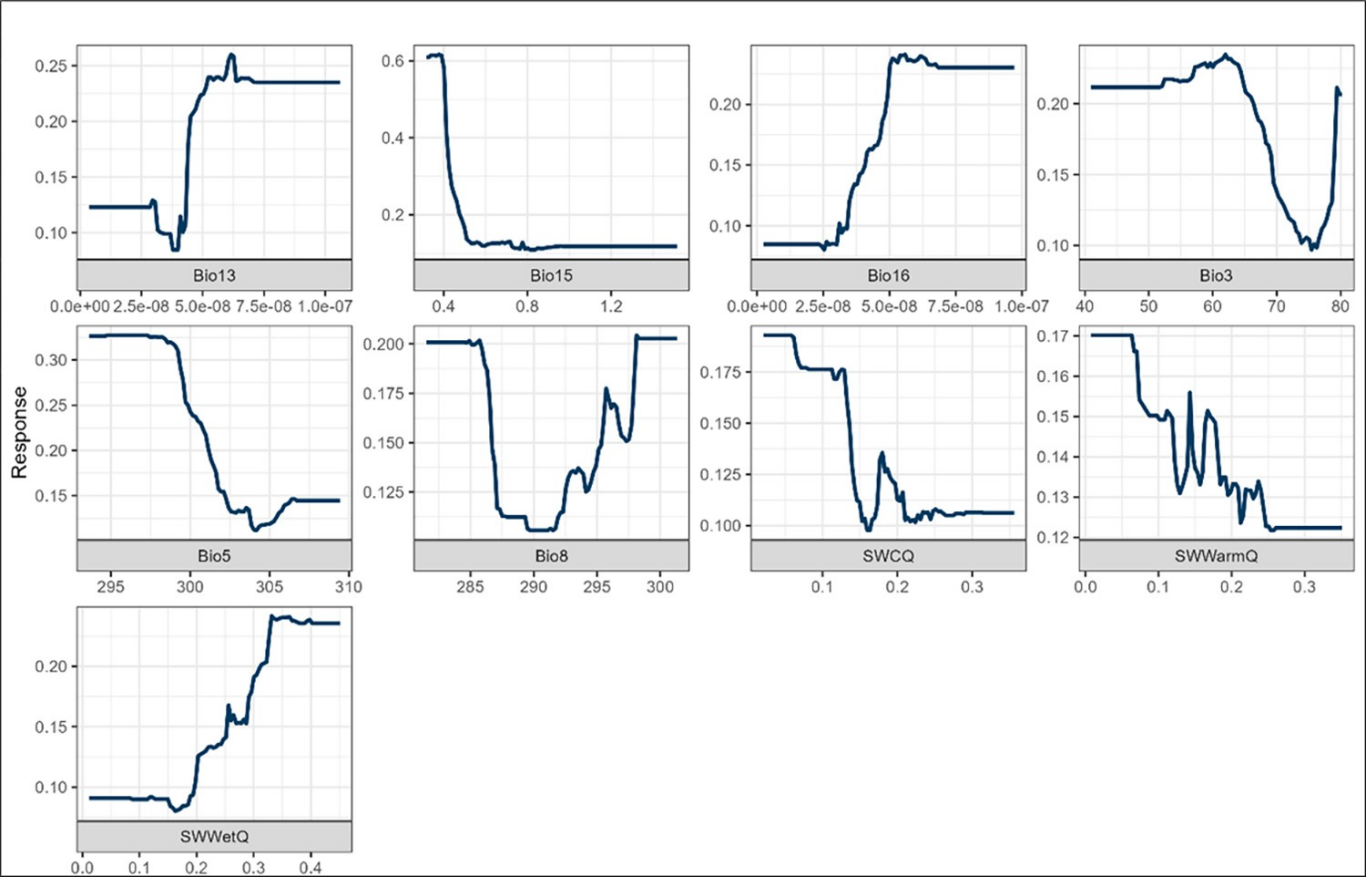
Response curves for *B*. *africanus* against bioclimatic variables. (Bio13 ms^-1^, Bio15%, Bio16 ms^-1^, Bio3%, Bio5 K, Bio8 K) and soil variables (SWCQ m^3^ m^-3^- Soil water for the coldest quarter, SWWarmQ m^3^ m^-3^- Soil water for the warmest quarter and SWWetQ m^3^ m^-3^- Soil water for the wettest quarter).

Among the three models that modelled *B*. *africanus*, precipitation-related variables had the greatest influence on the suitability for schistosomiasis, indicating that most *B*. *africanus* species thrive in rainfall environments, as shown by the response curves (Fig 5) and in (S1–S3 Figs). De Kock et al. [11] concluded that most *B*. *africanus* species occur in regions with 300 mm—700 mm/a of rainfall. For the GLM and MaxEnt, precipitation for the wettest quarter (bio16) (November to January) and precipitation for the wettest month (bio13) accounted for 65% in determining the suitability of schistosomiasis. Soil water for the wettest quarter contributed (32%) to the suitability for schistosomiasis. A study by Adekiya et al. [16] found that rainfall patterns are associated with the prevalence of schistosomiasis. Moderate precipitation allows for the transportation of snails and supports the creation of new habitats and temporary snail habitats [62]. Temperature variables contributed 59% to schistosomiasis suitability. *Bulinus africanus* is better adapted to colonising cooler environments. Appleton [63] concluded that cooler temperatures contributed the most to *B*. *africanus*. De Kock et al. [64] found that the optimal temperature for *B*. *africanus* reproduction was between 23°C and 26°C. Based on these results, *B*. *africanus* survived the longest at lower temperatures. Isothermality (bio3) (6.1%) did not affect schistosomiasis suitability. In the RF model, bio 16 accounted for 35.5%, followed by soil water (32%). Combined temperature variables contributed 31.9%, while the mean temperature of the wettest quarter (bio8) contributed 13.3% to the suitability of schistosomiasis in SA. This indicates that *B*. *africanus* thrive in both cold and warm climates. Thus, *B*. *africanus* suitability depends primarily on precipitation, temperature, and seasonal variations rather than annual averages. This is because the environment in SA varies significantly from season to season, and the parasites that cause schistosomiasis require specific conditions to survive and thrive [65, 66]. Thus, precipitation, temperature, and seasonality are critical factors in determining the suitability of the environment for these parasites.

**Fig 6:**
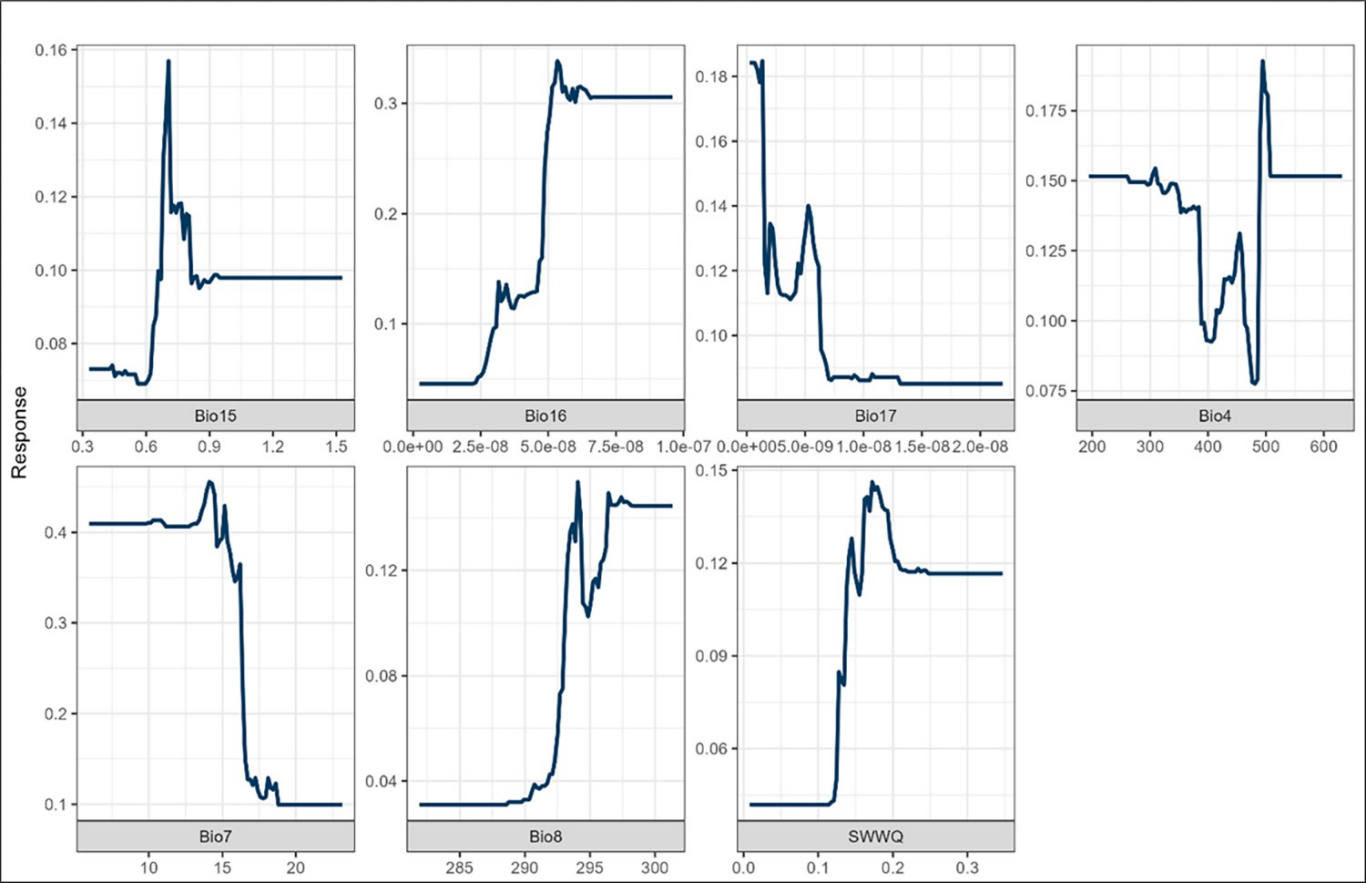
Response curves for *B*. *globosus* against bioclimatic variables. (Bio15%, Bio16 ms^-1^, Bio17 ms^-1^, Bio4 K, Bio7 K, Bio8 K, and soil variables SWWQ m^3^ m^-3^- Soil water for the wettest quarter).

*Bulinus globosus* distribution is significantly influenced by temperature and precipitation variables in all three models, as shown in (S4–S6 Figs). *Bulinus globosus* accounted for 41.7% of the suitability of infected snails by using precipitation in the driest quarter (bio17) and bio16, as shown in (Fig 6). A study by Pennance et al. [67] found that schistosomiasis-transmitting snails are more abundant during post-rainy seasons and more likely to transmit the disease. Madsen et al. [8] conducted a study on schistosomiasis along the lake shore of Malawi and reported that *B*. *globosus* is prevalent in post-rainy seasons or early winter. Due to bio16, heavy rainfall can cause floods, resulting in snail species migrating to new habitats. Therefore, potential habitats are created, allowing schistosomiasis-transmitting snails to migrate actively to nearby favourable habitats [68]. Temperature-related variables accounted for 38.3% of snail suitability. Similarly, *B*. *africanus*, bio 8 contributed the most (19.8%), followed by the annual temperature range (bio7) (Max Temperature of Warmest Month—Min Temperature of Coldest Month) (9.3%). The response curves for *B*. *globosus* show an increasing trend in bio8 (Fig 6). Therefore, *B*. *globosus* prefers hotter conditions with less moderate rain than *B*. *africanus*. In the MaxEnt model, temperature variables were the major contributors to *B*. *globosus’* suitability. Bio7 contributed 22.6%, followed by bio18 (18.4%). In contrast, Qin et al. [69] studied *Thuja sutchuenensis* and found that bio18 was the most suitable, followed by bio14 and bio6. According to the results from the current study, Similar to GLM and MaxEnt, the temperature annual range in the RF was the most influential variable in determining the suitability for *B*. *globosus*. A study by Joubert et al. [70] concluded that *B*. *globosus* is more resistant to high temperatures than *B*. *africanus*. There has been an increase in temperatures in SA, especially in tropical areas like Limpopo and Mpumalanga. Consequently, it could lead to an increase in the abundance of *B*. *globosus* species in the future. Joubert et al. [70] showed that *B*. *globosus* could withstand temperatures (34° to 40°C). Shiff et al. [71] concluded that *B*. *globosus* could survive in thermally harsh environments as they are relatively high in increasing temperatures.

**Fig 7:**
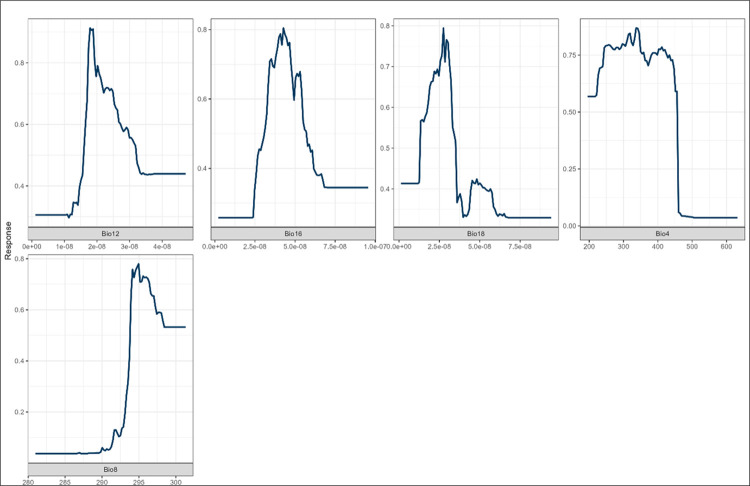
Response curves for *B*. *pfeifferi* against bioclimatic variables. (Bio12 ms^-1^, Bio16 ms^-1^, Bio18 ms^-1^, Bio4 K and Bio8 K).

In modelling *B*. *pfeifferi*, results showed that temperature seasonality (bio4) (standard deviation ×100) contributed the most (47%) in all three models, as shown in (S7–S9 Figs). The GLM and MaxEnt models showed that *B*. *pfeifferi* prefers a climate with more variability in temperature throughout the year. As a result, the snail is provided with the temperature fluctuations it needs to thrive [65]. Based on GLM and MaxEnt, bio4 accounts for 46.9% of *B*. *pfeifferi’s* suitability. The response curves for bio12 and bio16 show that *Biom*. *pfeifferi* do not favour moderate to high rainfall (Fig 7). Manyangadze et al. [21] studied schistosomiasis in the Ndumo area of the uMkhanyakude district and found that *B*. *pfeifferi* were suitable in the cold and dry seasons (winter) following the rainy season. Moodley [65] concluded that *B*. *pfeifferi* was influenced by winter minimum temperatures. Christensen et al. [72] found similar results, in which *B*. *pfeifferi* bred extensively following rainy seasons. It was found that bio18 accounted for 44.8% of snail suitability. A study in Ethiopia [73] showed that moderate rainfall is instrumental in increasing schistosomiasis by accumulating sufficient surface water in ponds. During heavy rainfall events, water levels rise, causing water turbulence. As a result, flow rates may increase, disrupting snail habitats and reducing cercariae survival [73]. The response curve for bio8 shows that *B*. *pfeifferi* favours temperatures higher than 290K (16.8C°) (Fig 7). The RF model also showed that bio4 (22.9%) and bio8 (15.5%) were the highest predictors of this snail’s suitability. This is because bio4 and bio8 help determine the climate conditions the snail prefers. Bio4 is an influential factor affecting *B*. *pfeifferi’s* climate suitability. In SA, *B*. *pfeifferi* is marginally suited to the climate because of variations in night and day temperatures and high daytime temperatures (30°C). There was a notable difference in temperature tolerance for *B*. *pfeifferi* compared to *B*. *globosus*. It is pertinent to note that despite being widely distributed throughout the country, *B*. *pfeifferi* snails are not tolerant of high temperatures [24]. Furthermore, a study by Sturrock [74] showed that *B*. *pfeifferi* mortality was high at 32°C. In addition, Deka [1] and Young [52] found that bio3 and bio4 had a higher relative contribution to modelling *Biomphalaria straminea* distribution. This might be because *B*. *pfeifferi* and *B*. *straminea* belong to the same genus, suggesting these species are likely affected or influenced by similar environmental factors. Studies by McCresh et al. [41, 42] have shown that seasons are integral to schistosomiasis distribution and transmission.

### Ecological niche models

**Fig 8:**
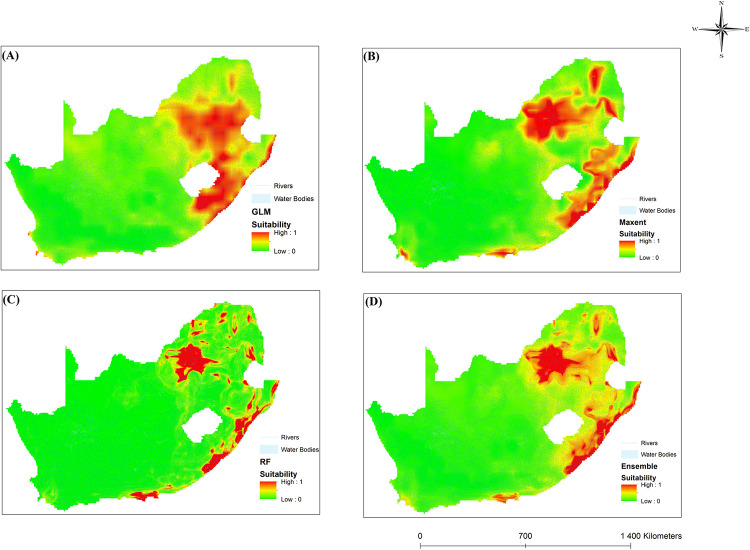
Areas where bioclimatic variables favour *B*. *africanus* distribution in South Africa using four different models, namely (A) GLM, (B) MaxEnt, (C) RF and (D) Ensemble.

**Fig 9:**
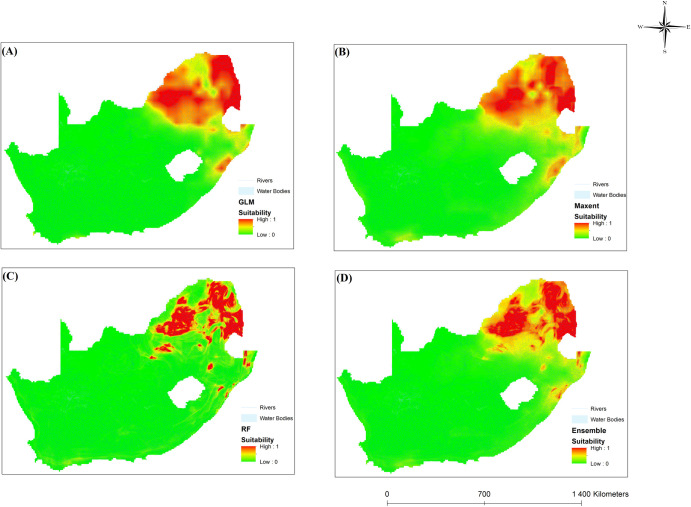
Areas where bioclimatic variables favour *B*. *globosus* distribution in South Africa using four different models, namely (A) GLM, (B) MaxEnt, (C) RF and (D) Ensemble.

**Fig 10:**
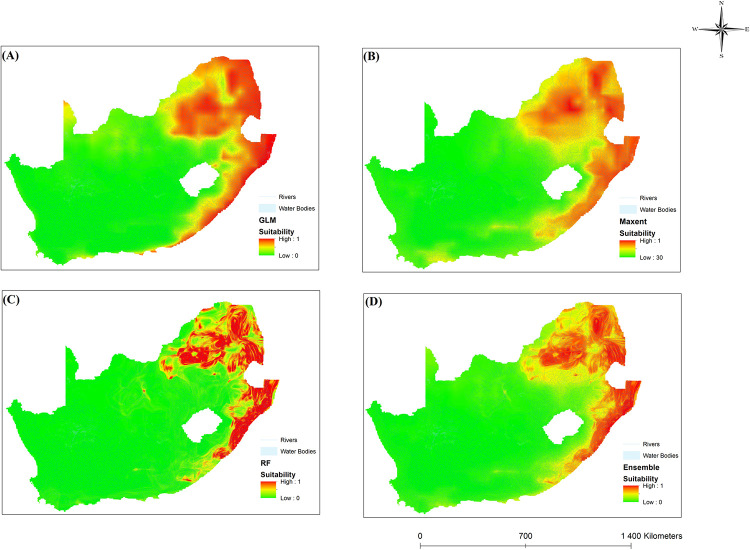
Areas where bioclimatic variables favour *B*. *pfeifferi* distribution in South Africa using four different models, namely (A) GLM, (B) MaxEnt, (C) RF and (D) Ensemble.

The ecological models are reasonably accurate at predicting the distribution of intermediate hosts based on bioclimatic variables. Precipitation and temperature are known factors in the development of intermediate snail hosts. Climate variability is integral to determining geographical distributions and is expected to alter species patterns [75]. According to the results, temperature and rainfall can influence the distribution of schistosomiasis in SA. The results of this study were supported by [65]. Figs 8–10 illustrate the results for the three chosen ENMs. Red shows highly suitable areas, whereas yellow depicts moderately suited locations. Green shows areas that are not suitable for schistosomiasis transmission. Additionally, snail presence probability varied with location, indicating the varying suitability of sites for *B*. *pfeifferi*, *B*. *globous*, and *B*. *africanus*.

*Bulinus africanus* was distributed in the eastern part of Gauteng province, parts of Mpumalanga and the North West Province (Fig 8). *Bulinus africanus* was found to be widespread west of the Eastern Cape province. *Bulinus africanus* is better adapted to colonising cooler environments and cannot tolerate high temperatures [65]. The mortality of intermediate hosts may be due to temperature increases [23]. This is likely due to the species’ tolerance of extreme environments and adaptability to environmental changes [65, 76]. *Bulinus africanus* can survive in areas with temperatures (of 15° to 30°C) and moderate rainfall, making it well-suited to its distribution range. The GLM, MaxEnt and the ensemble model showed that parts of the Western Cape were suitable for transmitting schistosomiasis (Fig 8). Even though ecological models predict suitable precipitation and temperatures for the Western Cape, no disease occurs under these conditions. Appleton [77] concluded that the natural acidity of waterbodies such as rivers and dams in this province could negatively affect intermediate hosts.

Fig 9 shows that *B*. *globosus* is distributed in the eastern areas of SA, particularly in the Limpopo, Mpumalanga, Gauteng, and eastern regions of the North West Province. The distribution extends to the coastal areas in KwaZulu-Natal. It is likely because *B*. *globosus* requires hotter temperatures and moderate rainfall [65]. *Bulinus africanus* was commonly found in the eastern regions of SA and to the south of Humansdorp. *Bulinus globosus* and *Bul*. *africanus* is sensitive to dry conditions and is closely related to temperature [23]. The *Bulinus* group is influenced by temperature and water flow [78]. Most *B*. *africanus* individuals were found at lower temperatures than *B*. *globosus*. This may be because *B*. *africanus* thrives in cooler climates [77, 79]. With increasing temperatures, *Bul*. *globosus* is more likely to survive than *B*. *africanus* [80]. In this way, *B*. *africanus* species can colonise habitats on the highveld of SA and Gauteng. Parts of the Western Cape also have suitable environments for transmitting schistosomiasis (Fig 9). The GLM shows Cape Town to be ideal for schistosomiasis transmission, while MaxEnt, RF, and ensemble indicate the southern Western Cape as moderate suitability. Despite the favourable climate conditions for schistosomiasis, it does not occur in parts of the Western Cape, likely due to high salinity and acidity levels in rivers and dams of the province [76].

It was found that *B*. *pfeifferi* was more abundant and distributed spatially across SA than *B*. *africanus* and *B*. *globosus*. Hence, most *B*. *pfeifferi* observations were found across SA. The spatial distribution of *B*. *pfeifferi* is like that of *B*. *globosus*. *B*. *pfeifferi* can be seen distributed in the northern and coastal areas of Kwa-Zulu Natal and around the coast of the Eastern Cape (Fig 10). The distribution covers most of Gauteng, Limpopo and Mpumalanga. *B*. *pfeifferi* preferred warmer temperatures with average rainfall [72]. As a result, *B*. *pfeifferi* and *B*. *globosus* are characterised by similar temperatures and rainfall patterns in the same regions. The differences in *B*. *globosus* and *B*. *pfeifferi’s* distribution are due to the required rainfall and temperature thresholds these species can tolerate. *Bulinus globosus* prefers high temperatures, whereas *B*. *pfeifferi* prefers warmer temperatures and is found in post-rainy seasons [65].

Fig 10 illustrates that the Northern Cape has an increased potential for transmitting schistosomiasis, such as the Kgalagadi Transfrontier Park. This could be due to the ideal temperature and rainfall conditions for intermediate hosts in this region. However, no snail vectors were found or sampled in the Northern Cape. Moodley [65] concluded that no disease occurred in the Northern Cape, but the temperature in the province was suitable for schistosomiasis.

In our study, ensemble models perform better than individual ENM models, as reported in other ecological studies. In the United States, ensemble models found potential habitats for three types of fish [81]. According to the ensemble models created in the present study, the most suitable areas for *B*. *africanus*, *B*. *globosus* and *B*. *pfeifferi* were found in the eastern and coastal regions of SA. The most suitable areas were the tropical and rural areas of SA, also located close to freshwater bodies. These findings agree with those reported in [81] regarding high schistosomiasis infection rates in villages near lakes. Results from ENM showed that temperature and precipitation were essential factors in snail distribution. This finding agrees with [73] that reported variations in rainfall and temperature could affect schistosomiasis transmission.

Based on our study, we found that temperature and rainfall can have an impact on the distribution of schistosomiasis in SA. Therefore, climate variability may influence the distribution of schistosomiasis in the future. This means climate change can affect schistosomiasis transmission, leading to shifts in the distribution of infected snails. If temperatures and precipitation levels change significantly, the geographical distribution and severity of schistosomiasis outbreaks could change.

## Conclusions

The present study aimed to use ENM to model the historical distribution of *B*. *africanus*, *B*. *globosus* and *B*. *pfeifferi*. Results showed that the ensemble models achieved the highest AUC scores (0.99) than the GLM, MaxEnt and RF. The ENM models showed that the distribution of *B*. *africanus* was found in the eastern and northern parts of Gauteng province, Mpumalanga, and the North West Province. *Bulinus africanus* species cannot tolerate high temperatures, and temperature increases could lead to the mortality of these intermediate hosts. *Bulinus globosus* was found in the northeastern areas of SA, particularly in the Limpopo, Mpumalanga, and Gauteng provinces and eastern regions of the North West Province. The distribution extended to KwaZulu Natal’s coastal areas with tropical and subtropical climates. This is likely because *B*. *globosus* requires warm temperatures and moderate rainfall. The spatial distribution of *B*. *pfeifferi* was similar to *Bul*. *globosus*, where *B*. *pfeifferi* was distributed in the northern and coastal areas of KwaZulu Natal province, extending to the coast of the Eastern Cape. The distribution covered most of Gauteng, Limpopo and Mpumalanga.

Although no schistosomiasis-transmitting snails are known to occur in the Western Cape due to the natural acidity of waterbodies that could negatively affect intermediate hosts, the ENM models showed a shift in the south and southwest regions of the Western Cape. This was likely due to the ideal temperature and rainfall conditions deemed suitable for schistosomiasis transmission in this province. Climate change/variability may lead to an end to schistosomiasis in SA and requires future research. The results from this study can be used to identify additional sites where Schistosoma-transmitting snails may already exist but have yet to be discovered. In addition, it is possible to identify future areas where the disease is likely to spread. This study contributes to the current understanding of schistosomiasis distribution in SA. The article updates the historical distribution of *Schistosoma haematobium* and *Schistosoma mansoni*.

## Supporting information

S1 Fig Relative variable importance for *B*. *africanus* using the GLM model. (PNG)

S2 Fig Relative variable importance for *B*. *africanus* using the MaxEnt model. (PNG)

S3 Fig Relative variable importance for *B*. *africanus* using the RF model. (PNG)

S4 Fig Relative variable importance for *B*. *globosus* using the GLM model. (PNG)

S5 Fig Relative variable importance for *B*. *globosus* using the MaxEnt model. (PNG)

S6 Fig Relative variable importance for *B*. *globosus* using the RF model. (PNG)

S7 Fig Relative variable importance for *B*. *pfeifferi* using the GLM model. (PNG)

S8 Fig Relative variable importance for *B*. *pfeifferi* using the MaxEnt model. (PNG)

S9 Fig Relative variable importance for *B*. *pfeifferi* using the RF model. (PNG)
