# Supplementary figures and images for: Modelling the historical distribution of schistosomiasis-transmitting snails in South Africa using ecological niche models
Source: PLoS One. 2023 Nov 30;18(11):e0295149. doi: 10.1371/journal.pone.0295149 (PMC10688899; doi:10.1371/journal.pone.0295149)

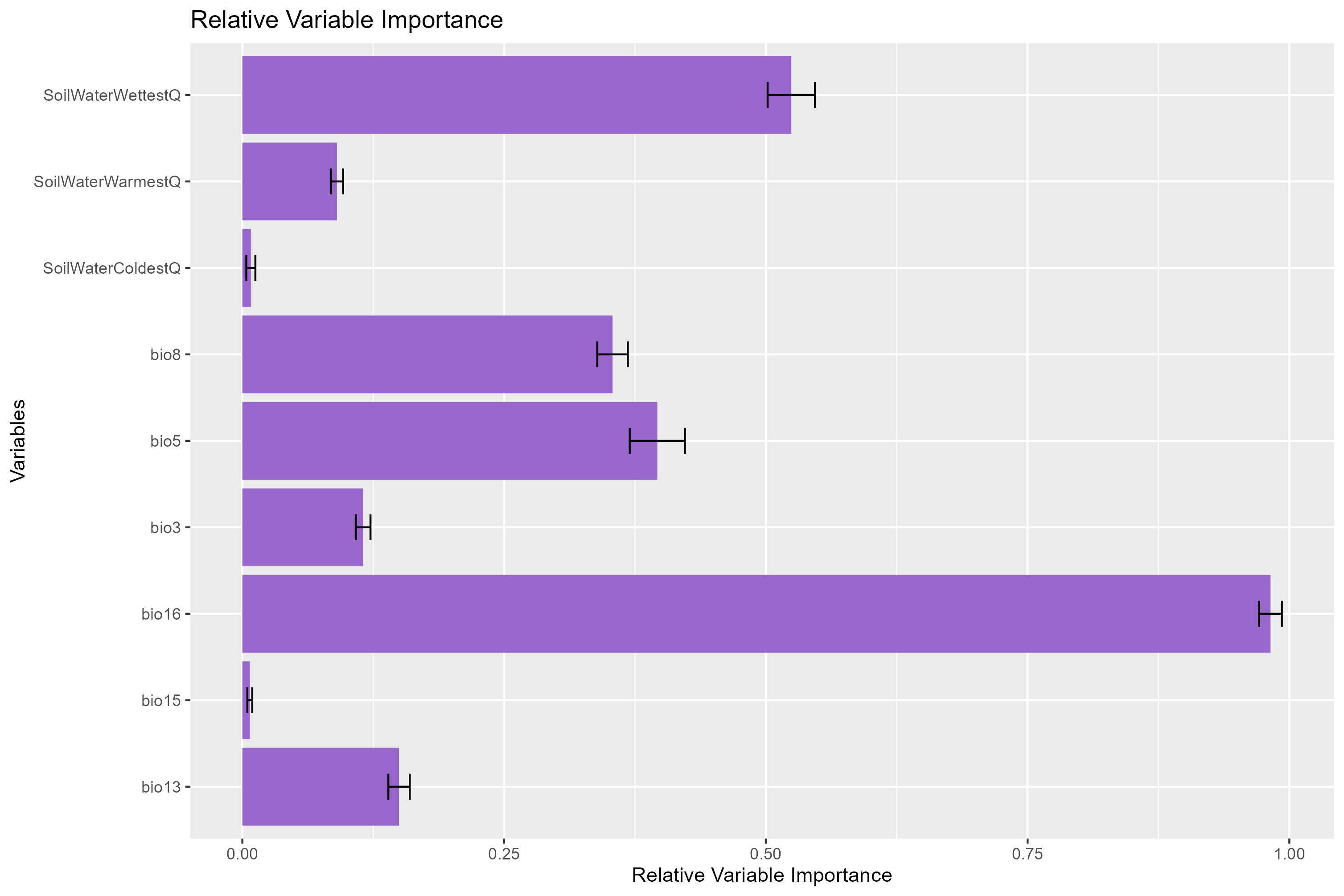

Supplement: S1 Fig — (PNG) [file pone.0295149.s001.png]

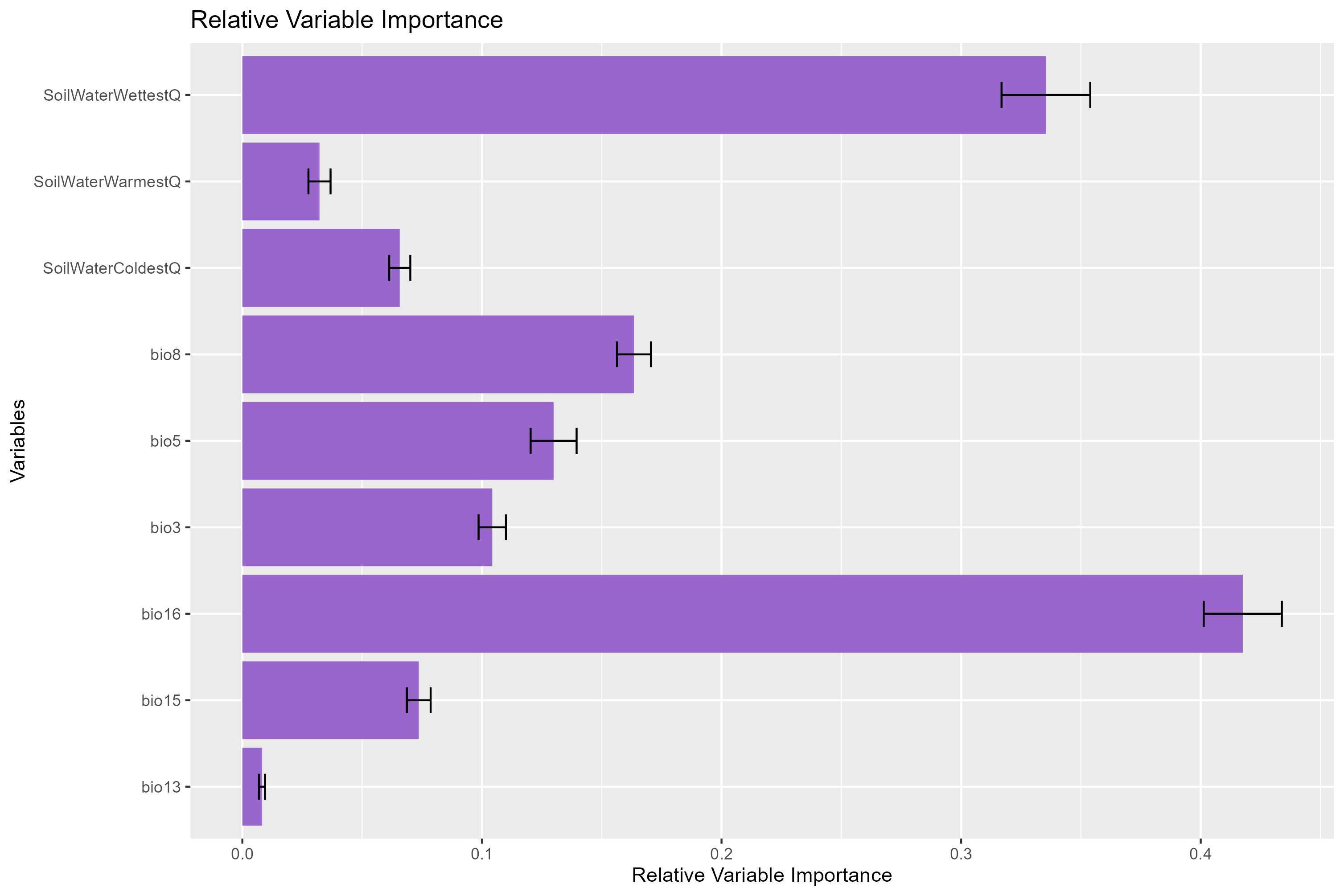

Supplement: S2 Fig — (PNG) [file pone.0295149.s002.png]

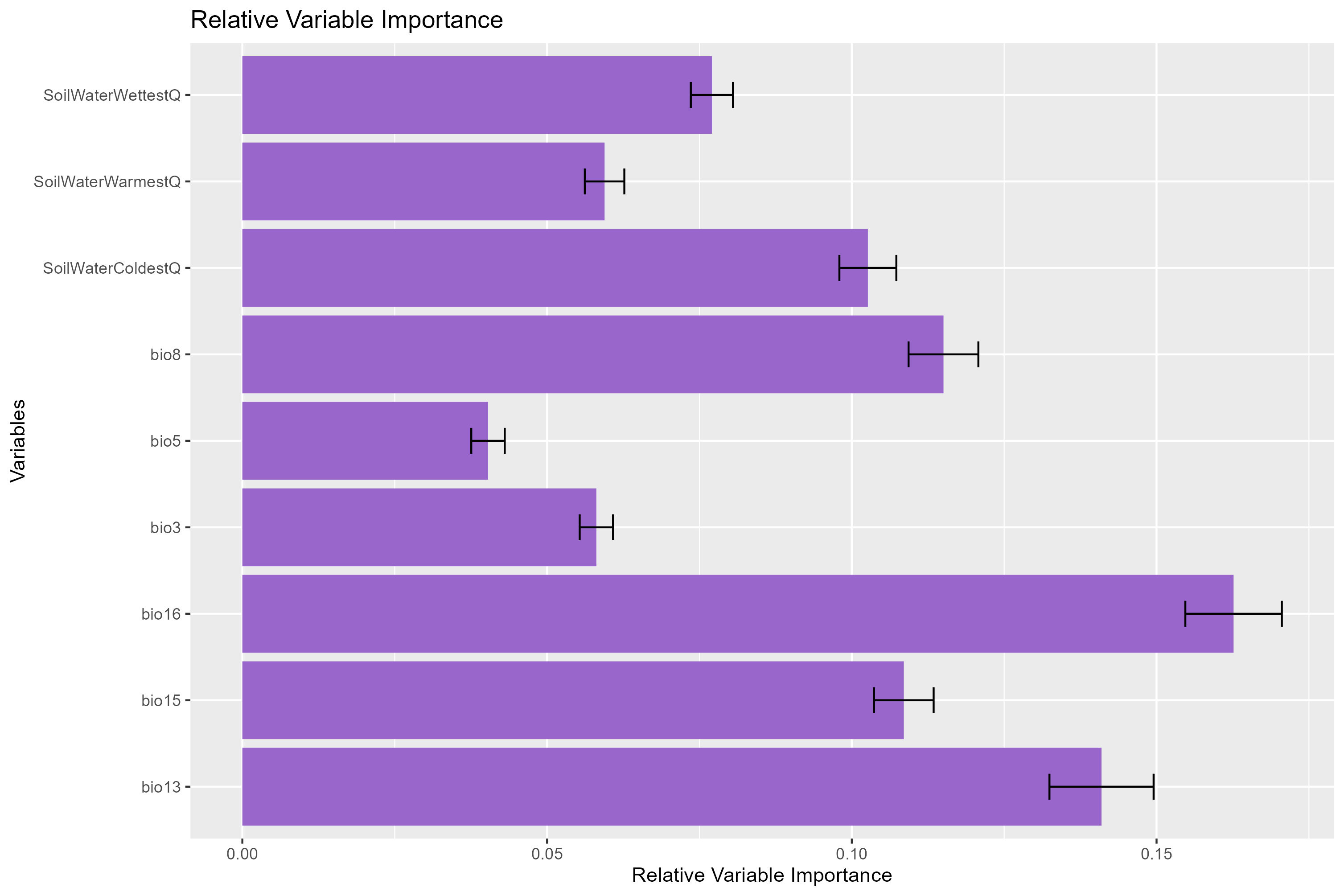

Supplement: S3 Fig — (PNG) [file pone.0295149.s003.png]

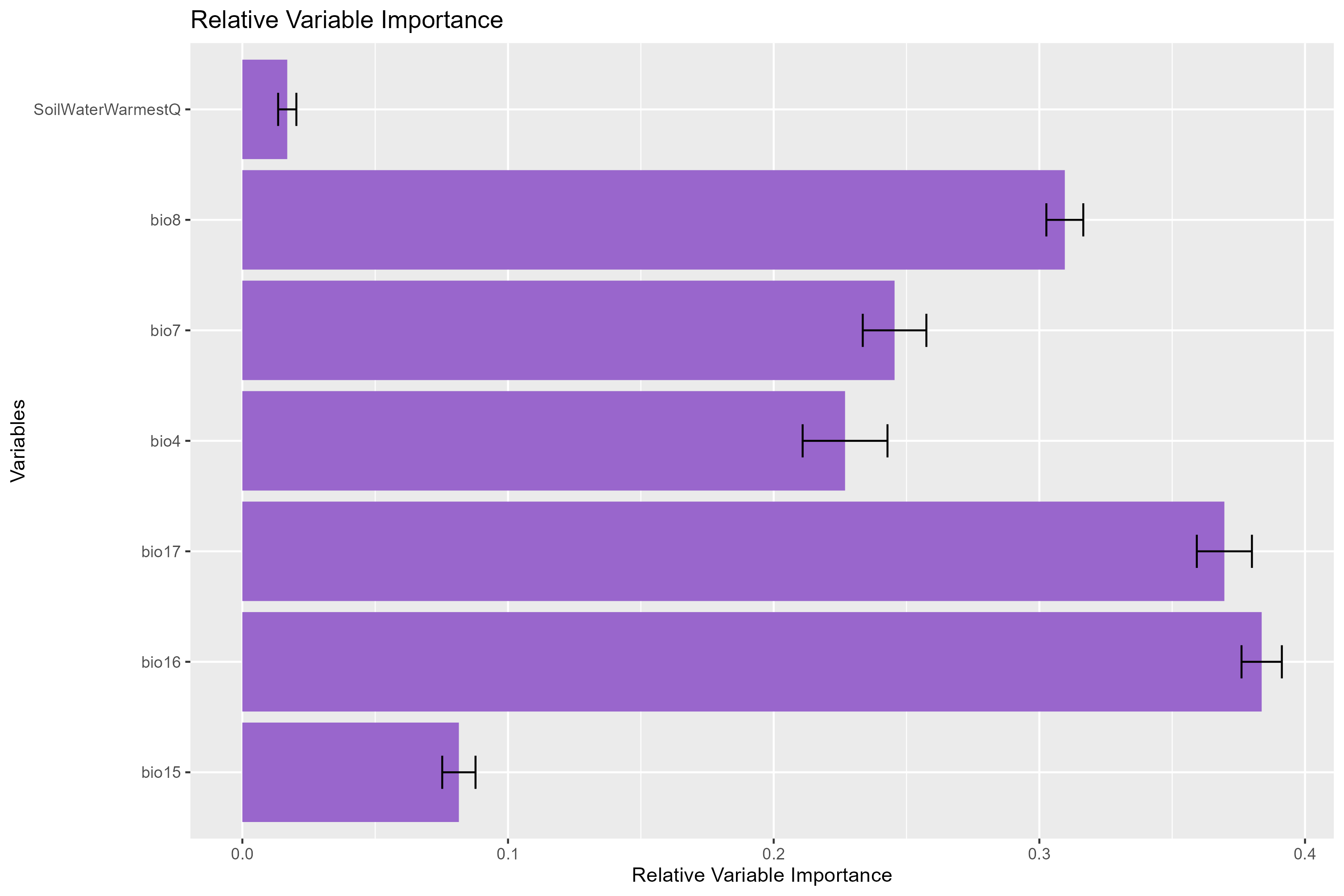

Supplement: S4 Fig — (PNG) [file pone.0295149.s004.png]

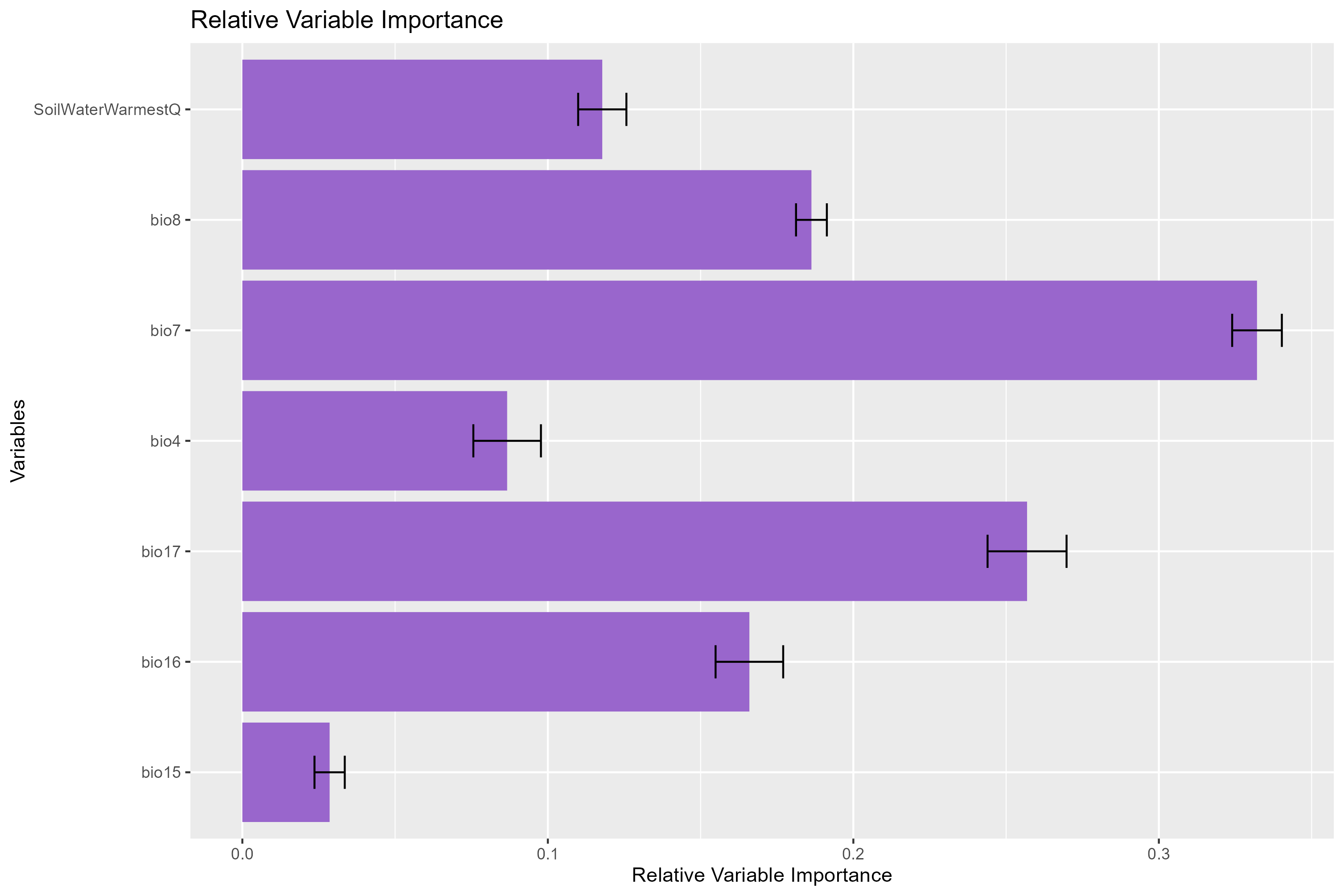

Supplement: S5 Fig — (PNG) [file pone.0295149.s005.png]

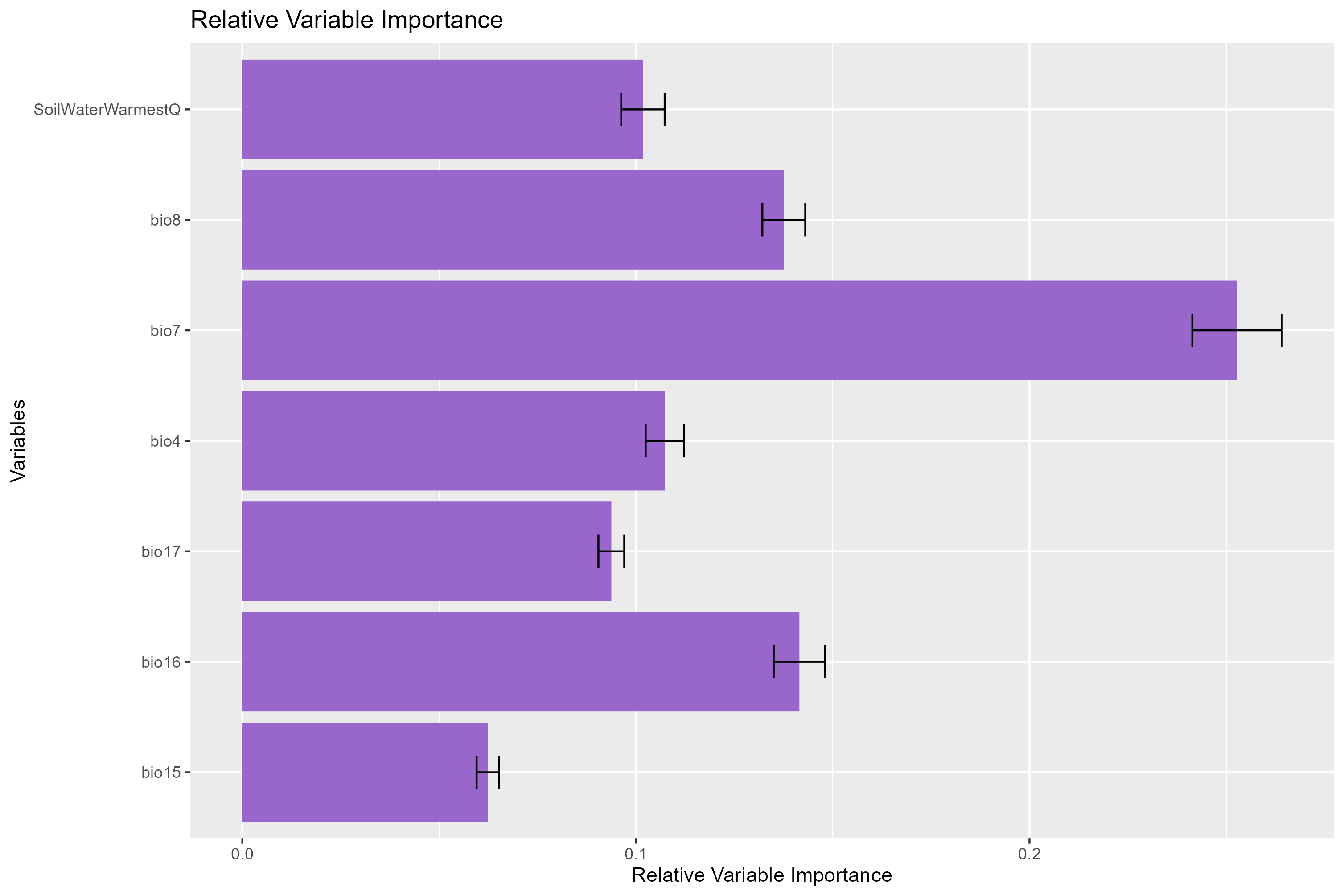

Supplement: S6 Fig — (PNG) [file pone.0295149.s006.png]

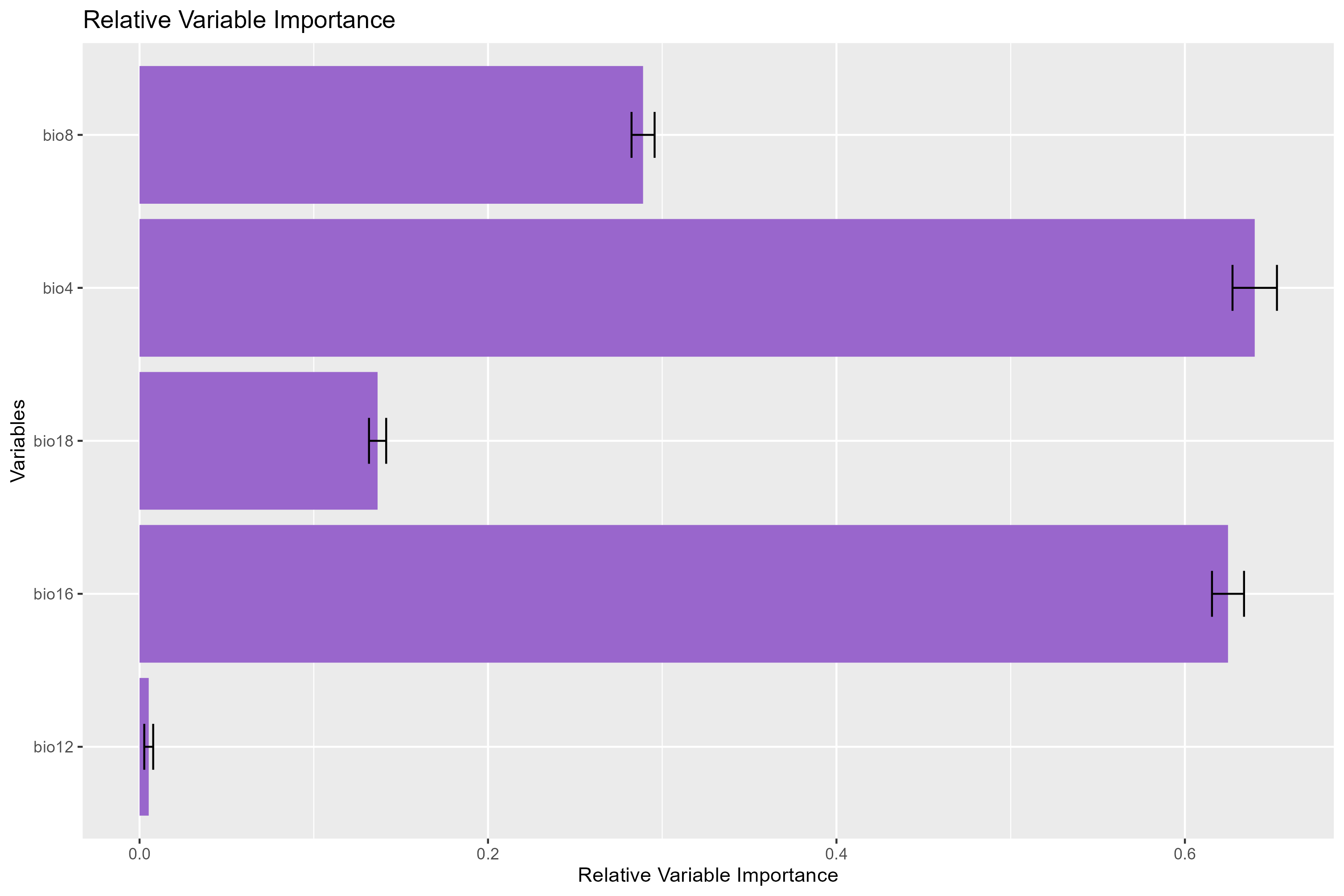

Supplement: S7 Fig — (PNG) [file pone.0295149.s007.png]

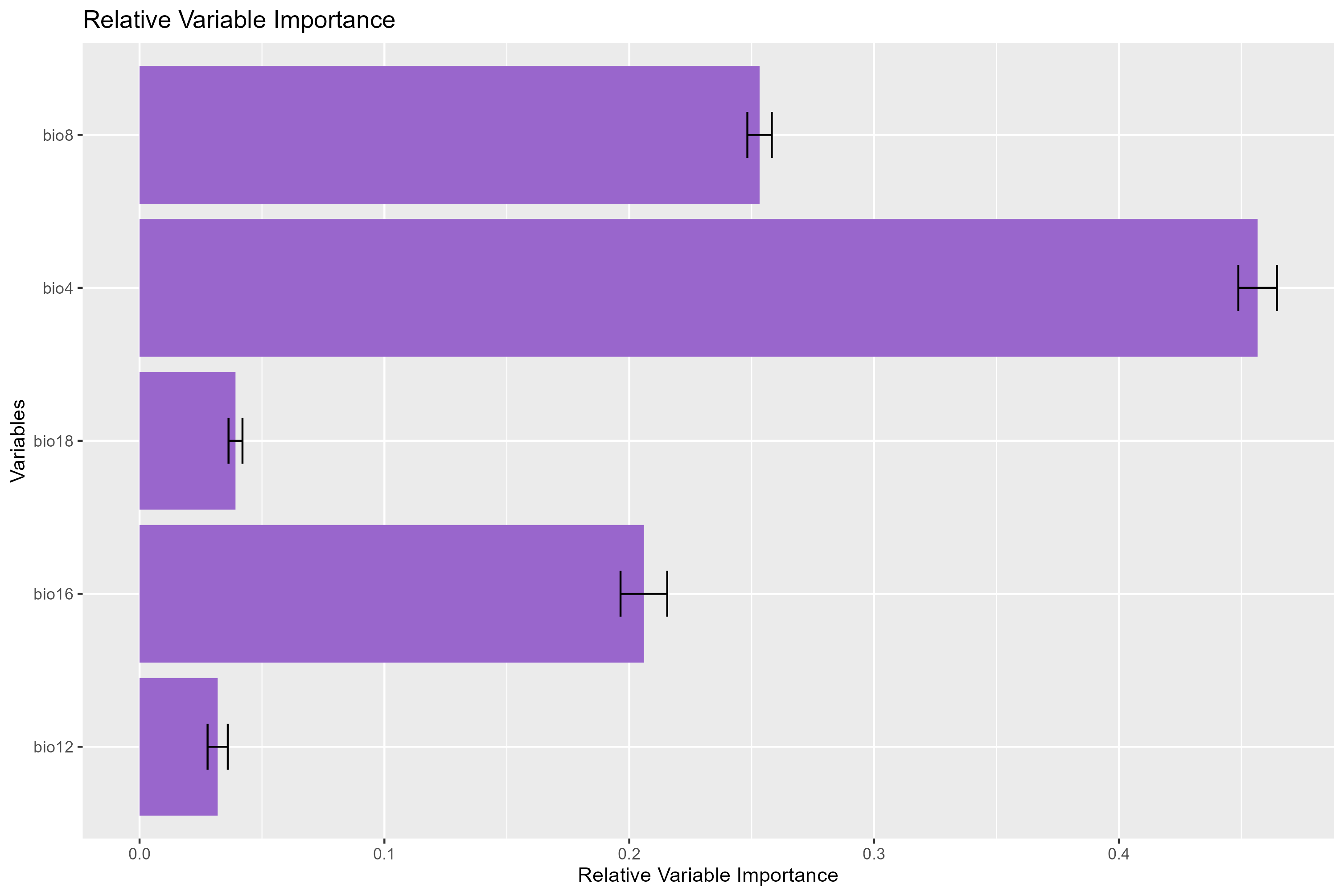

Supplement: S8 Fig — (PNG) [file pone.0295149.s008.png]

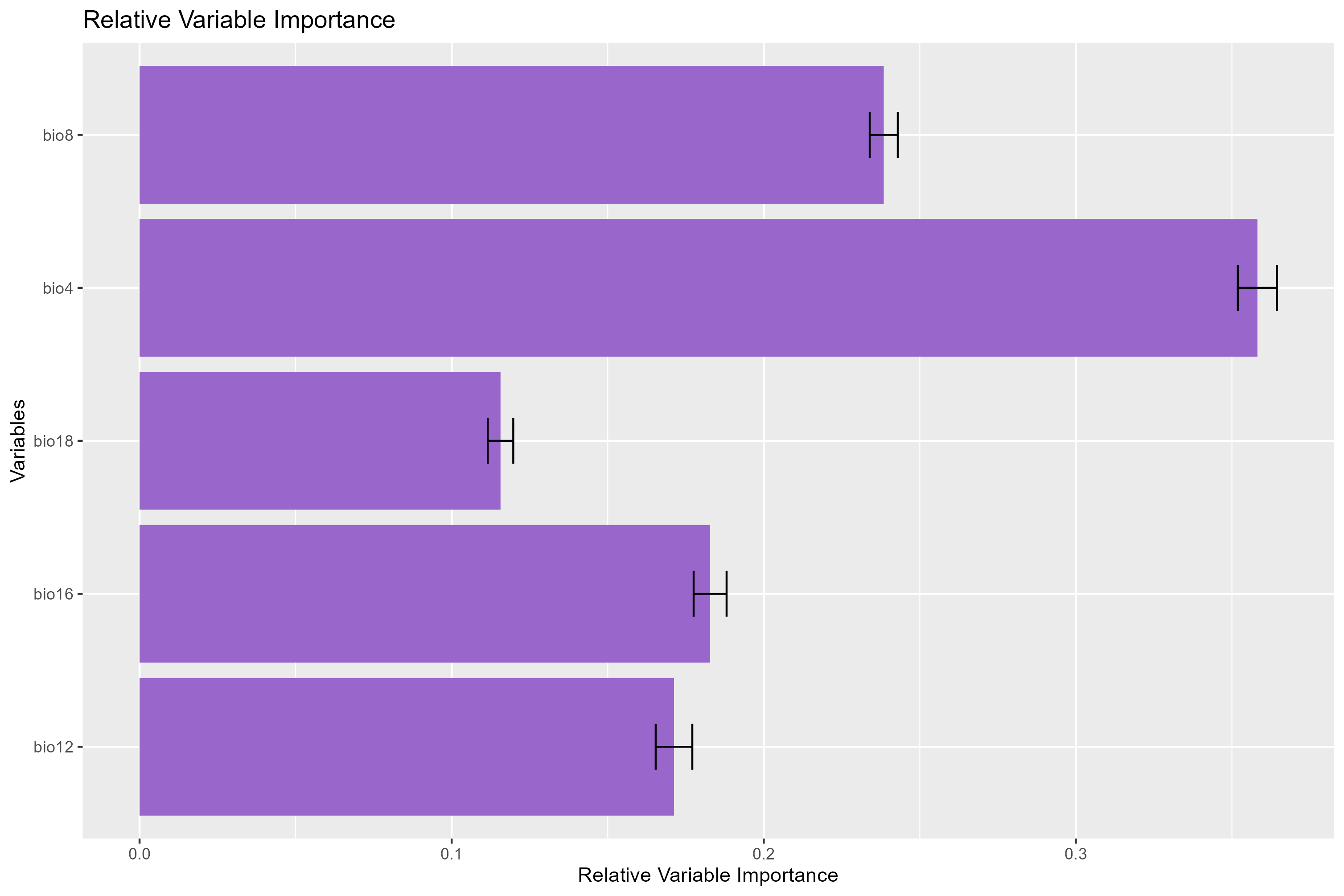

Supplement: S9 Fig — (PNG) [file pone.0295149.s009.png]
